# An Overview of the Modification Strategies in Developing Antifouling Nanofiltration Membranes

**DOI:** 10.3390/membranes12121276

**Published:** 2022-12-16

**Authors:** Nor Naimah Rosyadah Ahmad, Abdul Wahab Mohammad, Ebrahim Mahmoudi, Wei Lun Ang, Choe Peng Leo, Yeit Haan Teow

**Affiliations:** 1 Department of Chemical and Process Engineering, Faculty of Engineering and Built Environment, Universiti Kebangsaan Malaysia, Bangi 43600, Malaysia; 2 Chemical and Water Desalination Engineering Program, College of Engineering, University of Sharjah, Sharjah 27272, United Arab Emirates; 3 Centre for Sustainable Process Technology (CESPRO), Faculty of Engineering and Built Environment, Universiti Kebangsaan Malaysia, Bangi 43600, Malaysia; 4 School of Chemical Engineering, Engineering Campus, Universiti Sains Malaysia, Nibong Tebal 14300, Malaysia

**Keywords:** nanofiltration, antifouling, modification, membrane, water recovery, wastewater

## Abstract

Freshwater deficiency has become a significant issue affecting many nations’ social and economic development because of the fast-growing demand for water resources. Nanofiltration (NF) is one of the promising technologies for water reclamation application, particularly in desalination, water, and wastewater treatment fields. Nevertheless, membrane fouling remains a significant concern since it can reduce the NF membrane performance and increase operating expenses. Consequently, numerous studies have focused on improving the NF membrane’s resistance to fouling. This review highlights the recent progress in NF modification strategies using three types of antifouling modifiers, i.e., nanoparticles, polymers, and composite polymer/nanoparticles. The correlation between antifouling performance and membrane properties such as hydrophilicity, surface chemistry, surface charge, and morphology are discussed. The challenges and perspectives regarding antifouling modifiers and modification strategies conclude this review.

## 1. Introduction

It is undeniable that the world is facing increasingly severe problems with water and energy scarcity. More than 50% of the world’s population has severe water shortages, which are forecast to worsen in the coming years due to population growth [1]. This situation calls for urgent action to increase the clean water supply globally. Thus, the United Nations (UN) has established a target for water as part of the Sustainable Development Goals (SDG) agenda, namely Goal No. 6—“Ensure availability and sustainable management of water and sanitation for everyone” [2]. To accomplish this goal, desalination technology and water recycling and reuse can be implemented. 

Membrane technologies have become a possible option for increasing freshwater water supplies due to their low energy requirement, small footprint, clean process, and high productivity [3]. Nanofiltration (NF) membrane is one of the promising membrane processes for desalination and wastewater treatment. NF membranes typically have a molecular weight cut-off (MWCO) of 100–2000 Da with a nominal pore size of 1 nm, allowing them to separate monovalent salts and water from multivalent salts and low-molecular weight organics [4]. Moreover, the NF process possesses a good balance between energy usage and treatment capacity [5]. Despite the unique feature of NF and its advantages, fouling susceptibility is one of the challenges to the widespread implementation of NF membranes. In actual applications, the fouling problem is typically the most crucial aspect that may define the cut-off point of the membrane’s performance [5,6]. Membrane fouling can lead to a loss of water production and membrane integrity, lower water quality, and a shorter membrane life span [7]. Cleaning up the foulant to maintain the membrane’s performance usually involves more chemicals or energy, thereby increasing operational costs. As a result, the plant operation must bear a significant financial burden [7,8]. Therefore, it is essential to address the fouling issue to ensure the effectiveness of a membrane process in water reclamation applications, particularly in desalination, water, and wastewater treatment fields.

To alleviate the fouling problem, the research and development of antifouling membranes has become an important area. Compared to other fouling mitigation strategies, such as optimizing the operating conditions, the membrane modification approach to improve the antifouling feature is preferred because it will require only minor adjustments to existing setups [9]. Several reviews related to various NF aspects have been published thus far, including the NF application in water and wastewater treatments [10,11,12], overall NF process development and trends [13,14], ceramic NF [15,16], NF for resource recovery [17], NF for water recycling and reuse [3], and NF for dye/salt separation [18]. Concerns about the antifouling membrane topic have been reflected in some reviews, most of which focus on reverse osmosis (RO) membranes [19,20,21,22]. There are still no comprehensive reviews in the past five years that concentrate on the development of antifouling NF membranes.

This paper aims to present a current overview of recent scientific studies on developing antifouling NF membranes, focusing on modification strategies using three types of antifouling modifiers, i.e., nanoparticles, polymers, and composite polymer/nanoparticles. Unlike previous work [22], this review also includes the discussion on new emerging nanoparticles in NF modification, such as metal-organic frameworks, carbon quantum dots, porous organic frameworks, polyhedral oligomeric silsesquioxane, and their hybrid form with polymer materials. The review will begin with a brief description of the membrane fouling phenomenon, followed by a discussion on NF membrane modification approaches using various materials. The relationship between antifouling performance and membrane characteristics such as hydrophilicity, surface chemistry, surface charge, and shape are discussed. Finally, the perspectives associated with the advantages and limitations of each type of antifouling modifier and other aspects of modification procedures are provided. We also suggest research directions that require more investigation. This article gives an insight into new modifiers that have been extensively explored and provide an essential reference for constructing antifouling NF membranes by utilizing various nanoparticles, polymers, and composite polymers/nanoparticles.

## 2. Membrane Fouling 

Generally, a fouled membrane refers to a phenomenon where an undesired accumulation of solutes occurs either outwardly on the membrane surface, inside the pores (porous membrane case), or both [23]. Unlike low pressure-based membranes such as ultrafiltration (UF) and microfiltration (MF), which experience internal fouling, the NF membrane is primarily controlled by the surface fouling and scaling associated with divalent ions [22,24]. Fouling formation causes an increase in mass transfer resistance for water permeation, reducing membrane productivity. 

**Figure 1:**
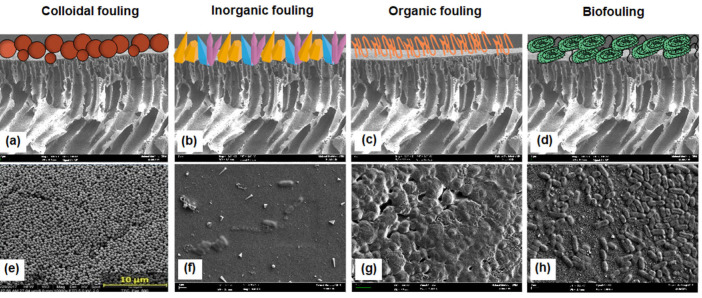
The schematic (**a**–**d**) and real membrane surface images (**e**–**h**) of different types of fouling (image (**e**) adapted from ref [32]).

Based on the nature of the foulants, it is possible to classify fouling as colloidal, inorganic, organic, and biofouling (Figure 1). Colloidal fouling occurs when the colloidal particles (size range of 1 nm–1 μm) adhere or deposit on the membrane surface [20]. Natural organic matter and organic and inorganic colloids, such as iron and silica, are examples of colloidal particulates. Since colloidal foulants can include both inorganic and organic elements, some sources classify colloidal fouling as part of either inorganic or organic fouling. Inorganic fouling (scaling) occurs when the level of inorganic ions in water exceeds the saturation point. This phenomenon leads inorganic ions to enter the nucleation step, forming the crystal that can deposit on the membrane surface or pores [25]. The most prevalent scalants on the membrane surface are inorganic salts with very low solubilities, such as barium sulfate (BaSO_4_), calcium carbonate (CaCO_3_), calcium sulfate (CaSO_4_), and silica (SiO_2_). Organic fouling describes the accumulation of organic matter on the membrane structure. Examples of organic matter include proteins, organic acids, nucleic acids, humic substances, and lipids [25]. Low- to medium-molecular weight organic foulants (300–1000 Da) have been observed to play a substantial role in the early phases of membrane fouling. On the other hand, high-molecular weight organic matter (> 50,000 Da) ruled the later stages of fouling layer development [26]. Biofouling, also known as proliferative fouling, involves the adhesion and proliferation of biologically active organisms on the membrane surface [27]. Biofoulants such as fungi, viruses, and bacteria create the biofilm layer. Moreover, the presence of salt in water or wastewater can stimulate the endogenous respiration and cell aggregation of bacteria, which in turn leads to an increase in the amount of extracellular polymeric substances (EPS) produced by cell secretion [28,29]. The high amount of EPS may cause the viscosity of the wastewater to rise even more and may also lead to the formation of an intractable cake layer on the surface of the membrane [30,31]. 

A recent study by Lin et al. [33] demonstrated the intricacies of the fouling phenomena occurring during the actual two-stage module NF process. It was found that at different locations of the modules, different types of fouling predominated. Biofouling and organic-biological fouling were found to dominate at the head of the first module, while organic fouling was predominant at the head of the second module. It was concluded that the type of fouling shifted at different locations in the module due to the continuous water quality change throughout the process. Thus, NF membranes must be able to cope with different fouling types for real water and wastewater treatment applications. 

Diverse strategies may be employed to reduce membrane fouling. Pre-treatment is a common method to minimize the foulants level in feed water before reaching the membrane unit. Some pre-treatment processes involve the feed pH adjustment and employ several techniques; either membrane (e.g., UF and MF) or non-membrane processes such as coagulation to remove pollutants that can exacerbate fouling [34,35,36]. In certain cases, anti-scalants can also be added to the pre-treated water before entering the membrane process but their amount needs to be regulated appropriately to prevent increased fouling accumulation caused by overdosing [37]. Besides feed water quality, hydrodynamic conditions associated with different crossflow velocities, specific transmembrane pressures, and other operating conditions such as permeate recovery rate could also influence the fouling phenomenon [7]. A membrane’s surface or pore interior can become fouled even if operating conditions and antiscalant dosage are optimised. Thus, membrane cleaning procedures such as chemical cleaning and physical cleaning (e.g., backwashing, reverse flush, and air spurge) have been implemented in the membrane process [7,23]. Even so, it is possible to lessen the frequency of these fouling mitigation measures by altering the membrane to make it less vulnerable to fouling. 

**Figure 2:**
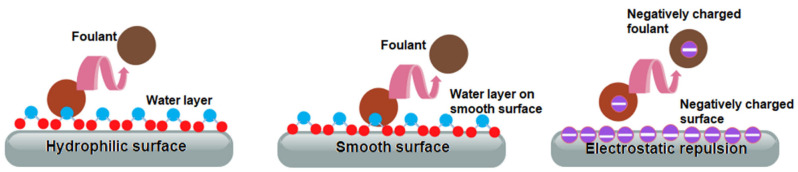
Illustration of membrane surface properties and antifouling mechanism.

A membrane with antifouling capability can be developed by tuning the membrane’s physicochemical properties to alter the membrane-foulant interaction. For instance, it is known that most foulants, including proteins, are hydrophobic by nature [38]. Thus, a hydrophilic membrane will have a lower fouling tendency since more water molecules could be adsorbed on the membrane surface, creating a hydration layer that could minimize the membrane–hydrophobic foulant interaction (Figure 2a) [20]. Typically, a hydrophilic surface is characterized by a low water contact angle (0° < Ɵ < 90°) and a higher value of Gibbs free energy [39,40]. Nevertheless, in practical applications, water contact angle measurement is considerably more frequently used for evaluating membrane surface hydrophilicity since it is simpler and easier to use. In terms of surface morphology, a smoother surface is less likely to become clogged with the foulant, whereas a rougher surface has a greater fouling propensity [41,42]. The fouling phenomenon can occur on thin-film composite (TFC) polyamide NF membranes despite their relatively hydrophilic surface. This is because the interfacial polymerization process used to fabricate the polyamide-based TFC often results in a rough membrane surface [23]. Therefore, membrane modification to reduce surface roughness can enhance the antifouling property. Meanwhile, tuning the surface charge is another approach to fabricating an antifouling membrane [22]. The fouling can be minimized by improving the electrostatic repulsion between the charged membrane surface and charged foulants. Hence, adjusting the membrane surface charge should consider the charge characteristics of targeted foulants in the water or wastewater to be treated. For instance, the negatively charged membrane is anticipated to have superior anti-biofouling efficacy since most bacteria are negatively charged at neutral pH [43]. 

## 3. Strategies for the Development of Antifouling NF Membrane

Generally, two common strategies have been identified for fabricating the antifouling NF membrane, namely (i) blending the additive or surface modifying agents with membrane casting solution (for asymmetric membranes) or blending the additive with monomer during interfacial polymerization step (for thin-film nanocomposite (TFN)) and (ii) surface modification of pre-formed membrane. Coating, grafting, covalent coupling, plasma treatment, and surface patterning are examples of surface modification techniques that have been employed in improving antifouling properties [22]. The coating technique is a straightforward process in which a thin layer of material (e.g., nanoparticles, macromolecules, and polymers) is introduced onto the membrane surface without any covalent attachment. Solution coating or layer-by-layer (LBL) techniques can create a coating layer during surface modification. A curing procedure is sometimes applied to the coating layer to ensure stability. The grafting technique is conducted by adhering the surface-modifying polymers or macromolecules to the membrane surface via various grafting mechanisms such as redox, free radical, plasma enzymatic, cationic, anionic, and atom transfer radical polymerization (ATRP). Unlike grafting, the covalent coupling method uses low-molecular weight materials to adhere to the membrane surface via interaction with reactive functional groups. Meanwhile, surface hydrophilicity or surface crosslinking can be achieved by plasma treatment or irradiation procedures [22].

Various advanced materials have recently been used to modify the NF membrane to improve the antifouling properties. The following subsections discuss the recent updates in NF membrane modification strategies using different antifouling modifiers, which can be classified into nanoparticle, polymer, and composite polymer/nanoparticle. 

### 3.1. Modification Using Nanoparticles

#### 3.1.1. Metal and Metal Oxide

Many significant efforts have been made to enhance the membrane’s self-cleaning capabilities and antifouling features. Among different approaches employed, introducing metal and metal oxide into the membrane matrix has been investigated in various applications. In general, metal and metal oxide nanoparticles (e.g., Ag, Cu, TiO_2_, Fe_3_O_4,_ and ZnO) possess biocidal and hydrophilic properties, making them promising antifouling agents [20,22]. Thus, the embedment of metal and metal oxide nanoparticles into a membrane can improve its physio-chemical properties and separation performance owing to the synergistic effect of the combination of components [44]. For instance, Soria et al. [45] used two different techniques, namely one-step co-deposition and two-step deposition methods, to attach nanoparticles of titanium dioxide (TiO_2_), zinc oxide (ZnO), and their mixtures to a polydopamine (PDA) surface on a commercial NF membrane. It was found that the addition of nanoparticles increased the membrane hydrophilicity by 25%. Moreover, the incorporation of 0.03 wt.% ZnO and a nanoparticles mixture (TiO_2_: ZnO) at different weight ratios, i.e., 1:2 and 2:1 via two-step deposition method, resulted in an NF membrane with antibacterial activity when tested using model bacteria (*Bacillus subtilis*). Even though both modification techniques did not significantly affect the MgSO_4_ rejection (∼95%), it should be noted that all the modified membranes containing TiO_2_: ZnO mixture exhibited a lower water permeability than the unmodified membrane. Increasing the nanoparticles loading on the membrane surface via a two-step modification method can increase the membrane resistance, reducing the water flux. Thus, proper control of nanoparticle concentration is essential to obtain optimum membrane performance. 

Although metal nanoparticles demonstrate outstanding antimicrobial behavior, the instability issue of the nanoparticles inside the membrane remains a concern. As an alternative, Qi et al. [46] modified the surface of the commercial NF membrane using three coating layers: PDA as the first inner layer, a mixture of hydroxyl propyl trimethyl ammonium chloride chitosan (HACC), chitosan, and chelated silver (Ag) nanoparticles as the second layer, followed by PDA coating in the third layer. The modified membrane showed a flux recovery rate of more than 96%, while an antibacterial test using *Escherichia coli* revealed no bacterial contamination on the membrane surface. This finding suggests that the three-layer modification approach can improve the antifouling feature and promote the antibacterial effect of the NF membrane. 

In another study, Ag nanoparticles were deposited into UiO-66-NH_2,_ and the composite nanoparticles (Ag@UiO-66-NH_2_) were then embedded inside the polyamide layer during the interfacial polymerization step [47]. The bovine serum albumin (BSA) solution filtration test revealed that the modified NF membrane could attain a flux recovery ratio (FRR) of 95.6%, indicating good antifouling properties. Increasing the Ag@UiO-66-NH_2_ loading to more than 0.03 g led to an antibacterial rate beyond 95% and enhanced the water flux to 47.3 LMH due to the porous nature of Ag@UiO-66-NH_2_ nanoparticles. Moreover, the rejection rate of Na^+^ salts decreased since the incorporation of Ag@UiO-66-NH_2_ caused a looser membrane structure. 

**Figure 3:**
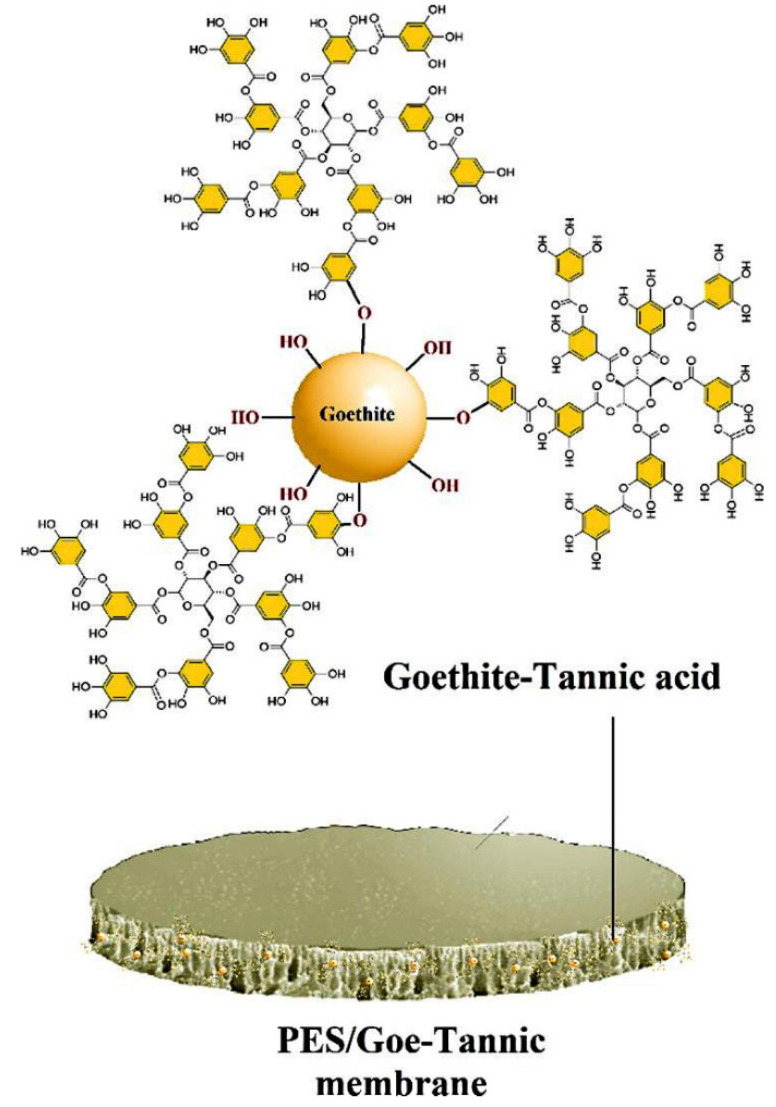
Illustration of Goe-tannic acid nanoparticles embedded in PES membrane [48]. (Copyright 2020. Reproduced with permission from Elsevier.)

Iron is a promising metal nanoparticle, but its high reactivity prevents its utilization as an antifouling agent [48]. As an alternative, the potential of iron derivatives such as iron oxy hydroxide for improving membrane fouling resistance has been explored. Goethite (Goe) is an example of iron oxy hydroxide that is widely available in the environment, inexpensive, highly stable, and possesses a high surface density of hydroxyl groups [48,49]. A recent work by Karimnezhad et al. [49] separately blended the Goe nanoparticle and its derivative, namely maleate ferroxane (Mf), with polyacrylonitrile (PAN) casting solution before the phase inversion process. The abundant carboxylic and hydroxyl group on Goe and Mf surface has improved the NF membrane hydrophilicity, water flux, and antifouling feature. Composite membranes with 0.5 wt.% Mf had higher dye retention and reversible fouling. In addition, the Mf nanocomposite membrane retained an acceptable performance (83.4–97% of FRR) for four cycles of powder milk fouling experiments. Subsequently, Saniei et al. [48] blended the Goe-tannic acid nanoparticles with polyethersulfone (PES) casting solution during the fabrication of the NF membrane via the phase inversion technique. The addition of 0.5 wt.% of the Goe-tannic acid nanoparticles has led to a nanocomposite membrane (Figure 3) with a smoother surface and antifouling properties, which is reflected by a high FRR (80.6%) compared to the neat membrane (43.7%). 

Another emerging composite metal oxide is transition metal ferrite nanoparticles such as cobalt ferrite (CoFe_2_O_4_). Cobalt ferrite has been extensively explored due to its chemical stability, high surface area, and selective adsorption characteristics [50]. Recently, Zareei et al. [50] fabricated the antifouling PES-based NF membrane containing cobalt ferrite-copper oxide (CoFe_2_O_4_/CuO) nanoparticles. Increasing the CoFe_2_O_4_/CuO loading up to 0.5 wt.% reduced the membrane surface roughness and decreased the water contact angle. This phenomenon consequently reduced the fouling propensity and enhanced the heavy metals removal (Cu^2+^, Ni^2+^, and Pb^2+^) of nanocomposite membranes due to the electrostatic exclusion of ions by the negatively charged membrane. However, increasing the CoFe_2_O_4_/CuO loading up to 1 wt.% can result in particle agglomeration, which reduces the effective surface area of nanoparticles and available adsorption sites, decreasing the heavy metal retention. ZnFe_2_O_4_ is another ferrite-based nanoparticle with high magnetic properties used to modify the membrane. Magnetic nanoparticles could augment fouling repellence under a magnetic field. Hence, ZnFe_2_O_4_/SiO_2_ nanoparticles were blended into the PES NF membrane to improve its antifouling property [51]. The surface roughness and pore size were increased under the magnetic field due to the accumulation of magnetic nanoparticles. The water flux was raised from 12 kg/m^2^ h to 38 kg/m^2^ h. Although the salt rejection of Na_2_SO_4_ dropped significantly below 70% after 2 h, as much as 86.0% flux could be recovered after being fouled by milk. Meanwhile, NF membranes incorporated with cerium oxide (CeO_2_/Ce_7_O_12_) nanoparticles were fabricated by Jamil et al. [52]. The antifouling test using humic acid (HA) foulant revealed that the NF membrane contained 1 wt.% cerium oxide, demonstrating the lowest irreversible fouling ratio (1.1%) due to the repulsion between the negatively charged surface and HA. The addition of cerium oxide has resulted in antifouling abilities and attaining the highest flux recovery after HA filtration without significant change in rejection (98%). Even though the incorporation of most metal or metal oxide nanoparticles has produced remarkable NF membrane features, the instability issues of the modified membranes, such as nanoparticles leaching, may prevent their application in industry.

#### 3.1.2. Carbon-Based Materials

Carbon-based nanomaterials such as fullerene, carbon nanotube (CNT), graphene, and carbon quantum dots are remarkable materials with unique properties that have been the center of attention in different areas of scientific research [53]. Membrane scientists have successfully incorporated carbon-based nanomaterials into membranes to form composite materials and acquire superior membrane properties [54]. The structure of most carbon-based nanomaterials consists of layers of sp2-bonded carbon atoms, each of which is connected to the three other carbon atoms by covalent bonds in the x–y plane. As a result, the sp2 carbon-based nanomaterials such as CNT and graphene are chemically inert, insoluble in most organic solvents, and naturally hydrophobic [55]. Therefore, to make carbon-based nanomaterials chemically active, soluble, and hydrophilic, these materials are mostly modified oxides and functionalized before application in membrane synthesis [56].

Researchers have used different functionalized single-walled (SWCNT) and multi-walled carbon nanotubes (MWCNT) to enhance the physicochemical properties of membranes [57,58]. For instance, Li et al. [59] have used carboxylated multi-walled carbon nanotubes (cMWNT) in the aqueous phase during the interfacial polymerization process of piperazine (PIP) and 1,3,5-trimesoyl chloride on the surface of polysulfone/non-woven UF membranes. The cMWNT-modified NF membranes have shown enhanced hydrophilicity (>50%), separation efficiency (>20%), pure water flux (>80%), and antifouling properties and showed more than 20% enhancement after three cycles. These observations can be attributed to the hydrophilic nature of the cMWNT and the changes in the microstructure and surface features of the polyamide layer after adding cMWNT [59]. It was observed that increasing the cMWNT content resulted in decreased membrane roughness. This favorable phenomenon, combined with hydrophilicity, enhanced the anti-fouling properties of the membrane as foulants are not prone to adhering to a relatively smooth and hydrophilic membrane surface; hence, the cMWNT enhanced the anti-fouling properties of the membranes [60,61]. 

In a similar study, Mahdavi et al. [62] used polypyrrole on raw and oxidized MWCNT (PPy-r and PPy-ox MWCNTs) to fabricate a thin-film NF membrane. The results have shown that the PPy-ox MWCNTs and PPY-r MWCNTs embedded membranes have 110% and 94% higher pure water flux in comparison to the unmodified membranes, respectively. The hydrophilicity of the membranes modified with PPy-ox MWCNTs and PPy-r MWCNTs was reduced by 17% and 12.5% compared to the raw membranes, respectively. This observation was due to the abundance of hydrophilic functional groups (hydroxyl and epoxy groups) of the ox MWCNTs. However, the fouling study using synthetic foulant solution (BSA) revealed that the PPy-r MWCNTs have 90% more fouling resistance than the raw and ox MWCNTs-modified membrane, which shows an almost identical fouling tendency to BSA. The authors have connected this phenomenon to the roughness of the ox MWCNTs membrane, which was observed in the FESEM images and was not quantified using AFM [62]. As a result, such a vast difference between the fouling results of the membranes is not justified scientifically.

**Figure 4:**
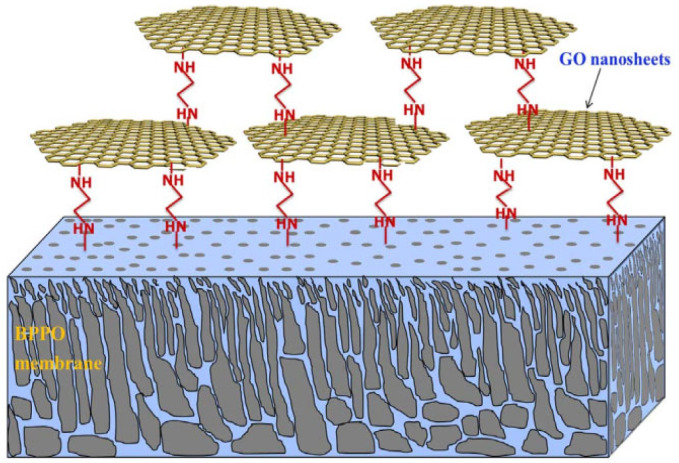
Illustration of low-pressure NF membrane embedded with EDA-functionalized GO [66]. (Copyright 2018. Reproduced with permission from Elsevier.)

Compared to CNTs, graphene-based materials have many advantageous properties, including excellent mechanical strength, large surface areas, excellent electrical and thermal conductivity properties, superior antibacterial properties, and lower prices [63,64]. As a result, many graphene-based materials have been used to fabricate membranes with improved separation, antifouling and antibiofouling properties. Graphene has been used to immobilize different types of metal and nonmetal nanomaterial in the structure of the membrane. The membranes modified with functionalized graphene material were reported to have a synergistic effect from both graphene and the decorated nanomaterial. Hence, these membranes have been shown to reduce the fouling and biofouling tendency of the membranes towards organic and inorganic materials [65]. For example, a low-pressure NF membrane embedded with ethylenediamine (EDA)-functionalized graphene oxide (GO) nanosheets (Figure 4) exhibited 50% increased salt rejection, enhanced fouling, and antimicrobial properties. Reduction in fouling and biofouling properties are directly correlated to the epoxy, hydroxyl graphene, and EDA nitrogen groups. On the contrary, the use of pure EDA in a membrane showed a detrimental effect on the antimicrobial and fouling properties of the membrane [66]. In another study, the addition of Ag-decorated GO to PES membranes enhanced the flux by 80%, hydrophilicity by 86%, and reduced roughness and fouling/ biofouling in a membrane bioreactor application. The enhanced antimicrobial and anti-biofouling of the membrane can be directly correlated to the dense and uniform dispersion of Ag-decorated GO nanoplates in the membrane matrix as the electrostatic repulsion of the GO combined with the oligodynamic effect of the Ag nanoparticles resulted in superior membrane materials with anti-biofouling capability. However, the high loading of Ag-decorated GO in a membrane produced flaws in the membrane matrix. Higher nanomaterial loading increased the casting solution’s viscosity, thus disturbing the thermodynamic stability and kinetics of the phase inversion process [67].

Carbon quantum dots (CQDs), which are also referred to as graphene/graphene oxide quantum dots (GQDs/GOQDs) and carbon dots (CDs), are the latest addition to the carbon-based nanomaterials family. CQDs have exhibited excellent optical and fluorescence properties. In addition, these materials have an abundance of carboxylic acid and epoxy groups, making them superhydrophilic. Due to their ultra-small size and ability to disperse better in solvents without agglomeration during nanocomposite membrane synthesis, CQDs have recently gained popularity over conventional nanomaterials for membrane synthesis [68,69,70]. CQDs have been blended in the membrane matrix to achieve improved performance and fouling/biofouling resistance. According to a study conducted by Koulivand et al. [71], incorporating CQDs into PES membranes improved the membrane’s stability and water permeability by more than 85%. Simultaneously, the membranes became highly resistant to fouling (40% less flux drop and 85% higher flux recovery after fouling cycles). This phenomenon can be attributed to the enhanced hydrophilicity and reduced surface roughness of the modified membranes [72,73]. Although carbon-based materials seem like a perfect candidate for membrane fabrication, they have some limitations, like any other nanomaterials, including a high cost of production, complex synthesis, reproducibility in the synthesis method, and poor control of the functionalization process. The combination of these problems prevented the carbon-based materials from reaching their potential in membrane modification. However, in the past few years, research in this area has been rapidly increasing and has indicated the strong potential for the application of these types of nanomaterial in membrane fabrication. 

#### 3.1.3. Metal-Organic Frameworks

Metal-organic frameworks (MOFs) are a novel type of nanoporous inorganic-organic hybrid materials with periodic network structures. MOFs are made up of metal ions that are connected with organic ligands. These materials have attracted increasing interest due to their unique characteristics, such as large specific surface area, tuneable pore structures, abundant adsorption sites, and great flexibility to combine particular species/functionalities without changing the framework topology [74,75,76,77,78]. In the past few years, MOFs have been increasingly incorporated during membrane synthesis to fabricate membranes with improved characteristics and performance for water and wastewater applications. This includes the utilization of MOFs as the main synthesis component or additive in the fabrication of NF membranes via various modification techniques. 

The strategy to incorporate MOF in the synthesis of TFN membranes and the functionalization of MOF play a crucial role in affecting the structures and properties of the membranes. Xiao et al. [79] demonstrated that adding MOF or amine-functionalized MOF to the aqueous or organic phase solutions will lead to the formation of a polyamide layer of different properties. The addition of pristine MOF in the organic phase produced a membrane with a higher water flux of 87.86 LMH compared to 46.31 LMH for the membrane prepared by adding MOF in the aqueous phase. This was because the MOF considerably slowed the interfacial polymerization rate when added to the organic phase, forming a thinner polyamide layer that reduced water transmission resistance during filtration. On the other hand, the presence of -NH_2_ groups significantly reduced the agglomeration of MOF in the aqueous phase compared to the pristine MOF, which improved the compatibility between MOF and polymeric matrix, enhanced the membrane filtration performance, and minimized the defects in the polyamide layer arising from nanofiller agglomeration. The membrane prepared by adding MOF in the organic phase was more hydrophobic compared to the membrane with MOF added to the aqueous phase. This led to the much better antifouling property of the membrane since it has a higher affinity to repel the hydrophilic BSA. The flux recovery rate still reached 96.82%, even after three fouling cycles with the BSA solution. 

The agglomeration issue of direct loading of MOF in membrane synthesis could also be minimized by another strategy proposed by Yang et al. [80]. The strategy started with preloaded Zn(II) cations as diffusion was slower during the interfacial polymerization on polysulfone support. It was found that the large nanopores formed during the interfacial polymerization process were self-sealed by an in situ-grown zinc imidazole framework (ZIF) in a counter-diffusion polymerization reaction of Zn(II) cations with imidazole ligands. Consequently, the interphase defects found in typical TFN membranes incorporated with ex situ-synthesized MOF were reduced. The membrane modified with in situ-grown ZIF attained much better antifouling properties and recorded a low permeance decline ratio and high permeance recovery ratio during BSA filtration. The performance of this membrane was close to the pristine TFC membrane, indicating that the defects formed on the polyamide layer due to modification with ZIF were minimal.

Nonetheless, most of the NF membrane studies utilized as-synthesized MOF due to the simplicity of fabrication steps. For instance, Golpour and Pakizeh [81] reported that the incorporation of MOF in the polyamide layer (through the organic phase) improved the antifouling properties of the membrane by enhancing the hydrophilicity of the membrane surface. The modified membrane achieved a higher flux recovery rate (97.8%) compared to the unmodified membrane (90.5%) when filtering kinetic hydrate inhibitor impurity. Liao et al. [82] went a step further in constructing hydrophilic hollow nanocubes (HHNs) in the polyamide layer by etching the zeolitic imidazolate framework (MOF) with tannic acid. The presence of a hydrophilic, hollow structure and negatively charged HHNs enhanced the membrane permeance by 190% compared to the pristine TFN membrane, which was higher than the improvement achieved by TFN membrane incorporated with MOF (116%). At the same time, the membrane with HHNs attained high FRR at 93.2% and 84.7% when filtering HA and BSA solutions, respectively. These performances were much better than pristine TFC and TFN membranes with MOF, possibly due to the adsorption of HA and BSA to membranes without HHNs. 

Lin et al. [83] functionalized MOF nanofillers with hydrophilic -NH/-NH_2_ groups and imidazole-2-carbaldehyde and incorporated it as an additive in aqueous monomer solution before undergoing interfacial polymerization to form a polyamide layer on polysulfone support. The presence of more hydrophilic functional groups promoted more crosslinking reactions and resulted in the formation of a more stripped polyamide surface with an enhanced surface area for the filtration process, as reflected by the elevated water permeability from 4.1 to 9.4 LMH/bar. Another factor that contributed to such improvement was the presence of MOF nanofillers, which offered additional transport channels for the permeation of water. In addition, the hydrophilicity of the modified membrane was also enhanced, leading to the improved antifouling property where the modified membrane attained a high residual flux of ~94% of the initial value after filtering BSA solution, compared to the pristine membrane at ~88%.

Lalabadi et al. [84] showed that the incorporation of magnetically modified MOF nanoparticles in the polymer matrix resulted in rapid solvent and non-solvent exchange during phase inversion, which gave rise to the increment in pore size. This, coupled with the enlarged pore size and enhanced hydrophilicity from 75 to 64° (water contact angle) corresponding to the pristine membrane and MOF membrane, resulted in the increment of flux from 8.1 to 28.5 LMH (operated at 3 bar). The improved hydrophilicity also contributed to the antifouling property of the modified membrane, which was reflected by 30% improvement in relative recovery rate (increased from 47 to 77%) when filtering BSA. A similar observation has also been reported where the incorporation of MOF in PES membrane led to higher FRR at 92%, compared to the pristine membrane at 62%, when filtering powder milk solution [85]. All of this indicated that the particles deposited on the membrane surface could be easily washed away through hydraulic cleaning for the modified membrane. The improvement could also be attributed to the smoother surface that prevented the accumulation of impurities on the membrane surface, as reported by Misdan et al. [86]. The membrane support layer incorporated with MOF possessed a smoother surface and exhibited 15–25% water flux reduction when filtering BSA solution. The flux decline was lower than the pristine TFC membrane, which suffered >40% flux decline. 

Apart from being used as an additive in the synthesis of TFN membrane, MOF can also be used as the main membrane fabrication component. Mozafari et al. [87] anchored a thin film of MOF on the surface of a chitosan-coated PES membrane to improve the surface properties as well as performance, especially antifouling. It was discovered that the membrane anchored with MOF showed a smaller reduction in the water flux when filtering bio-foulant (*E. coli*) and organic foulants (alginate and HA) compared to the membrane without MOF. The enhanced antibiofouling was ascribed to higher surface wettability and stronger antibacterial activity, which reduced the deposition of bacteria and inactivated the bacteria cells. The antifouling property was also improved due to the hydrophilic surface of the MOF-coated membrane, resulting in a lower propensity of organic matter to adhere to the membrane surface. This can be seen from the enhancement of flux recovery from 55 to 71% (alginate) and from 61 to 70% (HA) after the membrane was coated with MOF film.

**Figure 5:**
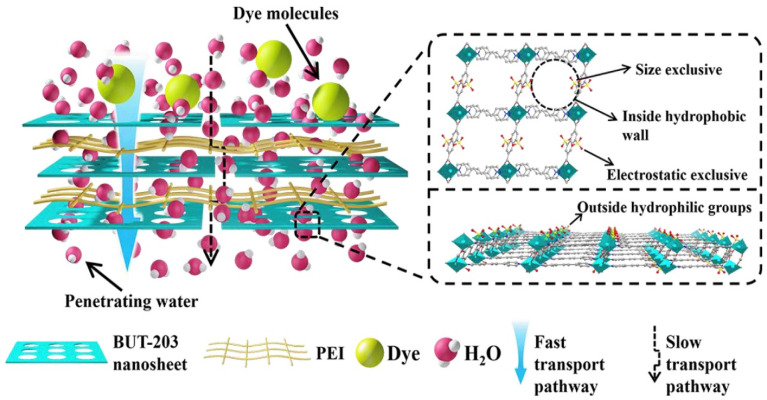
Proposed structure of modified membrane layers (MOF: BUT-23 nanosheet) [88] (Copyright 2020. Reproduced with permission from Elsevier.)

Shu et al. [88] spin-coated a mixture of MOF and polyethyleneimine on hydrolyzed polyacrylonitrile and observed that the water permeance was increased from 148 to 870 LMH/bar, corresponding to the addition of 0 and 73% loading of MOF in the membrane synthesis. Surprisingly, the significant permeability increase did not compromise much of the dye-rejection capability as the modified membrane still maintained methylene blue dye rejection at 97.9%. The authors attributed such findings (as depicted in Figure 5) to the lateral arrangement of MOF nanosheets on the membrane surface and the pore size of MOF, which was larger than water molecules but smaller than dyes. The combined features favored the passage of water molecules but rejected impurities of larger size. Furthermore, the membrane incorporated with MOF possessed a more negatively charged surface, which exerted stronger repulsion towards negatively charged impurities. As a result, the membranes regained 89% and 93% of fluxes when filtering BSA and HA solution, respectively, after the membranes were cleaned with deionized water. Such a finding suggested that the impurities could not firmly attach to the membrane and could be removed easily. Overall, various types of MOF have been used to improve the antifouling property of NF membranes. Nevertheless, the instability issue for most MOFs in aqueous solution remains a concern and needs to be addressed to unlock their potential in water or wastewater treatment applications. 

#### 3.1.4. Other Emerging Nanoparticles

Covalent organic frameworks (COFs) with porous and steady crystalline structures have also been used in the development of antifouling NF membranes. Gu et al. [89] produced COF from 1,3,5-tris(4-aminophenyl)-benzene (TPB) and 2,5-dimethoxyterephthaldehyde (DMTP) before blending into the polyamide membrane. The mesh morphology promoted the water permeability to 15.5 LMH/bar but still maintained a high rejection of Na_2_SO_4_ (98.9%). The fouling caused by BSA and NaCl was successfully reduced from 65.2% to 30.4% in terms of flux decline. Besides surface hydrophilicity improvement, the crumpled surface reduced foulant accumulation. Schiff-based network (SNW-1) COF was blended into PES membrane up to 2 wt.% [90]. The PES membrane with 0.5 wt.% of SNW-1 attained the highest rejection of Na_2_SO_4_, only 52.9%. The PES/SNW-1 membrane attained a water flux of 117 LMH, BSA rejection of 98%, and FRR of 88.9% due to the improvement of pore size and porosity. Ni et al. [91] reacted triformylphloroglucinol with hydrazine to form TpHz COF during interfacial polymerization. The polyamide membrane on the polysulfone UF support showed improvement in permeance of 23.6 LMH/bar with a rejection of Na_2_SO_4_ up to 96.9%. BSA fouling was reduced since the large pore of TpHz COF could reject BSA. Despite the improved antifouling property of COF-incorporated NF membranes, the issue regarding inhomogeneous size distribution of COF, which is detrimental to the synthesis of the uniform surface of polyamide films, needs to be addressed to promote COF applications in water treatment [89].

Polyhedral oligomeric silsesquioxane (POSS) nanoparticles offer porous cages and different functionality to improve NF membranes as they comprise the silicon-oxygen framework with a wide range of pendent organic groups. Sun et al. [92] introduced POSS through interfacial polymerization of PIP hexahydrate and 1,3,5-triethylbenzene (TMC) on PES support. Hydrophilic POSS such as polyethylene glycol (PEG)-POSS and octylamino (OA)-POSS enhanced NF performance. Activation using triethylamine (TEA) resulted in more interaction between primary amines and TMC. Hence, a permeate flux of 90 LMH with a Na_2_SO_4_ rejection of 98% was recorded after activation. The FRR after BSA fouling was improved as well due to the increased free volume and spatial structure of polyamide. 

**Figure 6:**
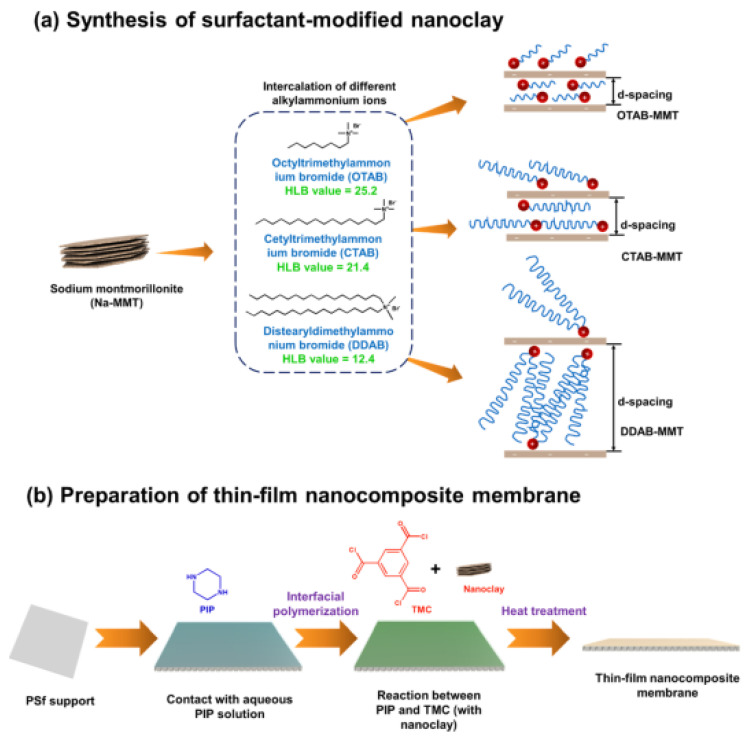
Illustration for (**a**) synthesis of surfactant-modified nanoclay; and (**b**) preparation of thin-film nanocomposite membrane [93]. (Copyright 2022. Reproduced with permission from Elsevier.)

Nanoclay such as montmorillonite (MMT) has also been used to fabricate the antifouling NF membrane. MMT with a negatively charged surface could be chemically modified before being incorporated into the membrane. For instance, MMT was modified with different types of cationic surfactants before being blended into the TMC solution (Figure 6) that was used to form the polyamide membrane [93]. Besides nanoclay dispersion, the d-spacing between nanoclays was enhanced by the long dimethyldioctadecylammonium bromide. A water flux of 66.02 LMH and a Na_2_SO_4_ rejection of 99.21% were attained. Moreover, the negatively charged surface was further enhanced to repel BSA electrostatically and result in stable permeability. Bentonite, an MMT clay originated from modified volcanic ash, can be transformed into an “electrically charged” particle when hydrated. In previous work, sulfonated bentonite improved the surface hydrophilicity of the polyamide membrane after blending [94]. Hence, the water permeability was enhanced beyond 10 LMH/bar. The hydrophilic network of bentonite reduced fouling by green oxalate dye and HA. The polyamide/sulfonated bentonite membrane rejected more than 98% of green oxalate dye, but it only removed about 20% of MgSO_4_. Meanwhile, hybrid nanomaterials such as nickel-bentonite nanoparticles (NBNPs) could be produced from the extract of Scrophularia striata through chemical conversion of metal salt into metal nanoparticles [95]. NBNPs were dispersed into the solvent during the preparation of PES dope solution, which subsequently formed an NF membrane through phase inversion. A water flux of 59 kg/m^2^ h was achieved by incorporating hydrophilic NBNPs. The membrane also rejected heavy metal ions such as Zn^2+^, Cu^2+^, and Pb^2+^ by more than 97.03%. FRR higher than 94% was maintained even during the filtration of milk.

Additional water permeation channels in a membrane could be created by incorporating Noria, a macrocyclic compound with 24 hydroxyl groups, six pendent cavities, and one large cavity at the center [96]. The polyamide membrane blended with Noria achieved water permeability of 147.6 LMH/MPa and Na_2_SO_4_ rejection of 98%. Even though increasing Noria concentration up to 0.8 w/v% can increase the membrane surface roughness, the enhanced surface hydrophilicity and negatively charge surface of modified NF membrane has prevented BSA foulant attachment. In another work, Dextran nanoparticle, a bacterial polysaccharide with great hydrophilicity, was also blended into the thin polyamide NF membrane [97]. The water permeability was improved by about 200% to 211.2 LMH/MPa without reducing the Na_2_SO_4_ rejection of 98.4%. BSA fouling was reduced and FRR of 80% was attained. However, excessive interfacial channels and internal pores created by the dextran nanoparticle could impair the rejection. 

In summary, numerous types of nanomaterials have been incorporated into NF membranes via different modification techniques (Table 1). Despite the enhanced fouling resistance capability of NF membranes, the nanoparticles’ agglomeration in the polymer matrix and poor compatibility between polymer and inorganic nanoparticles remains a major concern that need to be addressed since such phenomenon can cause deterioration in the membrane performance. 

### 3.2. Modification Using Polymers

**Figure 7:**
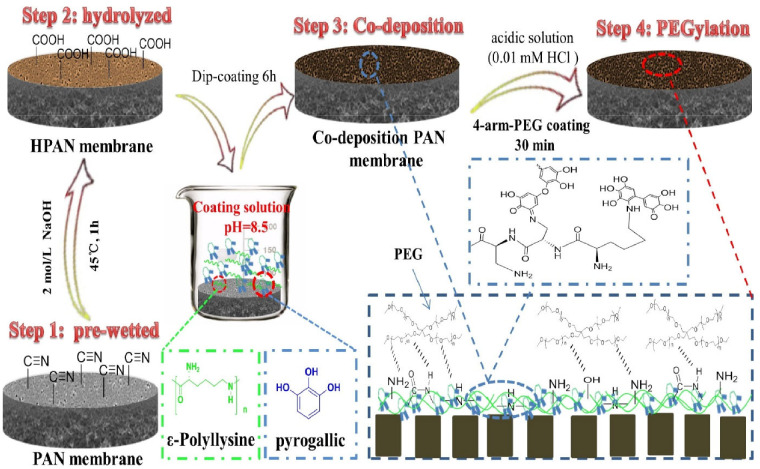
Preparation of a loose NF membrane involving ε-Poly-L-lysine and pyrogallic co-deposition and PEGlation steps [102] (Copyright 2018. Reproduced with permission from Elsevier.)

Membrane modification using organic polymers is another option to fabricate the antifouling NF membrane. Unlike inorganic nanomaterials, organic polymers possess a better compatibility between the polymeric chains, which is desirable for membrane surface modification. The application of a hydrophilic polymer layer on the NF membrane surface is one of the common methods to improve the fouling resistance of that membrane. Polyvinyl alcohol (PVA), polyethylene glycol (PEG), and PEG derivatives are examples of conventional hydrophilic polymers that have been used to modify NF membranes owing to their well-known antifouling capability [98]. PVA is a hydrophilic modifying agent which can be coated on the membrane surface or incorporated into the membrane during the interfacial polymerization process. Zhu et al. [99] compared two modification methods, namely gutter- and coating layer-assisted strategies to incorporate the PVA into TFC NF membrane. Results showed that that the PVA-coated NF membrane demonstrated an excellent antifouling behavior with more than 80% FRR during three cycles of filtration of HA. However, the water flux was slightly decreased due to additional resistance imparted by the PVA coating. Meanwhile, PEG polymer is commonly attached to the membrane surface via the grafting process, forming a polymer brush-like morphology [100]. Their antifouling properties are associated with steric hindrance and surface hydration [101]. The flexible long chains and large exclusion volume of PEG prevents large or hydrophobic molecule adsorption on the membrane surface [22]. The efficacy of PEG as an antifouling polymer is dependent on the surface grafting process and polymer design [98]. Consequently, a number of grafting techniques have been developed to enhance the efficacy of the PEG antifouling layer. For instance, Zhao et al. [102] modified the negatively charged hydrolyzed polyacrylonitrile (HPAN) substrate using ε-Poly-L-lysine (ε-PL) and natural material pyrogallic (PG) prior to reaction with four-arm PEG methoxy (4-arm-PEG) (Figure 7). The formation of the ε-PL/PG-mediated surface helps to improve the PEG binding on the loose NF membrane (LNF). The modified NF membrane showed a good antifouling performance with FRR of 85% during the long-term filtration test, which was conducted using real textile effluent. Moreover, it was found that the “pine-shaped” crystal clusters still remained on the membrane surface even after two and four cycles of washing, suggesting the stability of the modified NF membrane. A similar approach was reported by Ang et al. [103], who pre-modified the polyamide TFC membrane with glutaraldehyde (GA) prior to reaction with PEG. The presence of excess aldehyde groups can react with PEG hydroxyl groups, thus improving the fouling resistance of the NF membrane. The antifouling study which was carried out using 100 ppm BSA solution demonstrated that the PEG-GA-TFC membrane can attain a lower flux decline ratio (35.88%) and higher recovery ratio (99.09%) compared to that of the unmodified TFC NF membrane (37.08% and 89.34%, respectively). Despite the antifouling capability of PEG, it should be noted that PEG has poor stability due to the rapid autoxidation and degradation of PEG macromolecular chains during handling and storage at ambient temperature [104]. 

Besides hydrophilic polymers, dendritic polymer has also been used to improve the fouling resistance of NF membranes. This macromolecule possesses branched structure and a huge number of functionalities. Usually, dendritic polymers can be further classified into three subclasses, namely dendrimer, dendrigraft, and hyperbranched polymer [105]. Polyethyleneimine (PEI) is an example of dendritic polymer which comes in a wide variety of molecular weights. Recently, Zhang et al. [106] implemented the green rapid-coating (GRC) technique to develop an antifouling LNF based on crosslinked PEI and polyphenol-inspired capsaicin-mimic, i.e., N-(5-methyl acrylamide-2,3, 4 hydroxy benzyl) acrylamide (AMTHBA) for textile wastewater treatment. The negatively charged surface of modified LNF could prohibit the foulants from accumulating or blocking the membrane pores via electrostatic repulsion. This was reflected by the excellent antifouling property of AM-PEI/HPAN LNF, where the FRR of sodium alginate (SA), HA, and BSA aqueous solutions were increased from 81.2%, 94.8%, and 89.5% (in the first cycle) to 98.0%, 97.7%, and 98.1% (in the second cycle), respectively. The less preeminent fouling resistance for SA foulant could be due to the stronger interaction between residual hydroxyl groups on the LNF surface with the hydrophilic SA. Besides that, some researchers [107] introduced the phosphonate groups into PEI to generate a novel phosphorylated PEI (PEI- PO_3_Na) selective layer which provides the high permeability, anti-scaling and anti-fouling property to the loose NF due to the improvement in hydrophilicity, polarity, and chelation ability. The surface charge, pore size, and performance of the LNF membrane was tuned by varying the molecular weight of PEI-PO_3_Na from 1.8 to 70 kDa. The NF test, which was conducted using real industrial textile effluent, showed that the LNF with PEI- PO_3_Na 70 kDa possessed the best antifouling property since its smallest pore size can prevent the fouling induced by pore blocking in the case of LNF. Meanwhile, the presence of phosphate groups improved the antiscaling performance of PEI-PO_3_Na/PEI LNF membranes since the loosely packed structure of phosphonate-Ca^2+^ complexes could prevent the prenucleation and subsequent scaling. Unlike others that focused on hydrophilicity enhancement, Qian et al. [5] tried to improve the antifouling property by lowering the surface energy of the NF membrane. Their strategy involved the use of perfluorobutylsulfonyl-functionalized PEI (FPEI) as a monomer during the interfacial polymerization step to fabricate the fluorinated polyamide TFC NF. The surface free energy was reduced from 39.9 mJ/m^2^ to 31.1 mJ/m^2^ after incorporation of perfluoro groups, leading to a minimal interaction between membrane surface and foulants. Even though increasing the FPEI concentration increased the permeate flux of the NF membrane, excessive concentration (>0.75 wt.%) resulted in larger membrane MWCO and reduced the surface negative charge, lowering the Na_2_SO_4_ rejection (<50%). Apart from PEI, polyamidoamine (PAMAM) is another type of dendrimer that has also been used to fabricate antifouling NF membranes for heavy metal removal and desalination [108]. The large size of PAMAM provides a significant steric hindrance, while its abundant terminal amino groups can improve the membrane hydrophilicity. Previously, PAMAM was blended with annular supramolecular cucurbit [6]uril (CB6) and PIP monomer during the interfacial polymerization process to develop high-performance NF [109]. It is interesting to note that the modified membrane containing both PAMAM and CB6 not only exhibited a strong antifouling property in inorganic salt, but also in organic pollutant systems, suggesting its potential in broader applications. Even though most dendrimers can improve the fouling resistance of a membrane, its high production cost remains a concern [108].

Zwitterionic polymer is another class of a polymer that has been extensively applied to improve the antifouling property of NF membranes. Its polymer chain contains the same number of anions (e.g., sulfonate, carboxylate, and phosphonates) and cations (e.g., quaternized ammonium) [104]. Polycarboxybetaine, polyphosphobetaine, and polysulfobetaine are outstanding zwitterionic polymers due to their long-term durability, substantial hydrophilicity, and environmental stability [110]. It has been reported that sulfobetaine-based zwitterionic polymer has a seven-fold greater capacity to hold water molecules than ethylene glycol, demonstrating their superhydrophilicity [111]. Thus, zwitterionic polymers may be more effective in repelling biofoulants than PEG-based polymers due to their ability to form a thicker hydration layer on the membrane surface [112]. Moreover, electrostatic interactions enable the zwitterions with balanced charges and reduced dipoles to fully attract water molecules and repel charged proteins [113]. Previously, modification methods such as surface grafting and surface coating have been used to alter the surface of TFC NF with zwitterionic polymers. However, surface grafting is more frequently applied than the surface-coating technique. Ding et al. [111] used the surface-initiated atom-transfer radical-polymerization (SI-ATRP) to graft the zwitterionic sulfobetaine methacrylate (SBMA) from the PDA-modified NF membrane surface. The modified membrane, namely TFC-PDA-PSBMA, has demonstrated excellent antifouling performance in both simulated foulant solution and actual brackish water. This was reflected by the higher FRR value of the TFC-PDA-PSBMA membrane (90.8%) compared to the pristine TFC. Despite its promising antifouling property, the water permeability of the TFC membrane was slightly decreased after zwitterionic grafting. Nadizadeh and Mahdavi [110], who applied the surface-initiated reversible addition-fragmentation chain transfer (SI-RAFT) technique to graft the zwitterionic polymer on the TFC NF membrane, also observed the water flux reduction after membrane modification. Unlike the SI-ATRP method, the SI-RAFT technique is applicable for a wide range of monomers. Nevertheless, the zwitterionic grafting via surface-initiated radical grafting technique may form a thick layer on the membrane surface, increasing resistance to water permeation. Deng et al. [114] proposed another strategy to fabricate zwitterionic-based NF with antifouling features and high separation performance. In their work, the TFC NF membrane surface was first grafted with PEI, followed by zwitterionic functionalization via the quaternization approach. The NF with zwitterionic-PEI functionalities have demonstrated a thicker hydration layer than the control membranes (PEI-grafted NF and pristine NF). The thick hydration layer and enhanced steric hindrance have prevented the foulant’s adsorption on the zwitterionic PEI-membrane surface and suppressed its flux decline ratio to 21.8% and 40.2% when tested with BSA and lysozyme (LYZ) foulants, respectively. Moreover, the zwitterionic PEI functional layer in the sub-nanometer scale did not enhance the resistance to water permeation. The increase in water permeability (11.6% improvement) and ion selectivity (high divalent ion rejection; low monovalent ion rejection) was observed for the zwitterionic PEI-TFC NF membrane compared to the pristine NF membrane. In spite of the potential of zwitterionic polymers in antifouling surface engineering, it should be noted that some of the zwitterionic polymers possess challenges in terms of complicated synthesis procedures and high production costs [21,104].

PDA is a prominent biopolymer for membrane modification due to its capability to adhere to various substrates via hydrogen bonding, covalent bonding, and electrostatic interactions [115,116,117]. The PDA coating can improve membrane hydrophilicity since it contains catechol, quinone, and amino groups, making it one of the options for fouling resistance enhancement [20]. Even though PDA is hydrophilic, it has been demonstrated in some studies that the deposition of a single PDA layer on the NF membrane can significantly increase the membrane surface roughness [111]. Thus, in NF membrane modification, PDA is commonly integrated with other polymers or additives to improve the separation performance and antifouling properties [111,117,118]. For instance, Ding et al. [111] post-modified the commercial NF membrane with PDA first before subjecting it to zwitterionic functionalization. The role of the PDA layer on the NF membrane was to provide stable support for zwitterionic grafting since PDA could adhere well to the membrane surface via chemical bonding. Meanwhile, the zwitterionic grafting further reduced the membrane surface roughness and increased the membrane hydrophilicity compared to unmodified TFC and PDA-modified TFC. Similarly, Haresco et al. [117] also integrated the PDA and zwitterionic polymer during the fabrication of TFC NF with the antifouling property. In their work, a novel poly(dopamine-sulfobetaine methacrylate) [P(DA-SBMA)] was synthesized and incorporated into the polyamide layer of the TFC membrane during the interfacial polymerization step. The optimal membrane performance was observed at a 0.55 weight ratio (g of P(DA-SBMA) per g of TMC), where the modified membrane exhibited a pure water permeability of 73.11 LMH with 98%, 95%, 54%, and 42% salts rejection for Na_2_SO_4_, MgSO_4_, MgCl_2_, and NaCl, respectively. The antifouling test using BSA showed that a high flux recovery (99.53%) could be achieved by embedding the P(DA-SBMA) into the TFC, suggesting its antifouling potential. Meanwhile, another work deposited the PDA/PVA layer on the NF membrane to produce a membrane with both antifouling and chlorine resistance properties [118]. The presence of PVA contributed to the antifouling improvement, while the spatial structure of PDA increased the water permeance. The hybrid membrane showed a high FRR (98.9%) with a 6.1% of total flux decline ratio when tested with model foulant (HA). Overall, PDA has been commonly integrated with other polymers during NF membrane modification and imparts fouling resistance properties. Nevertheless, it has been reported that in some cases, PDA is susceptible to aggregation after long durations, which therefore reduces its stability [119,120,121].

In summary, several types of polymers (Table 2) have been used to modify NF membranes. Even though most of the polymers such as zwitterionic dan dendrimers successfully improved the antifouling property, the water permeability tends to decrease after membrane surface modification via polymer grafting or coating techniques due to additional mass transfer resistance. Hence, more improvement is needed in the polymer modification techniques to enhance the membrane performance.

### 3.3. Modification Using Composite Polymer/Nanoparticles

Several attempts have been made to overcome the particles’ agglomeration and the poor compatibility issue between inorganic particles and the polymer matrix. One of the approaches is to pre-modify or pre-functionalize the nanoparticles with a selected polymer before incorporation into the membrane. Moreover, such modification strategies can impart the synergistic effects of both polymer and nanoparticles, improving the separation performance and antifouling properties of an NF membrane (Table 3). Previously, biopolymers such as chitosan and cyclodextrin have been used to enhance nanoparticle dispersion in the polymer casting solution before phase inversion [122,123,124]. For instance, Bagheripour et al. [122] pre-modified the activated carbon (AC) nanoparticles with chitosan before blending them with PES casting solution at various particle loadings (0.05–1.0 wt.%). Increasing the chitosan-modified AC (ACh) loading up to 0.5 wt.% increased the water flux from 21 to 30 LMH. Even though the addition of modified AC reduced the pore size of the NF membrane, the effect of hydrophilicity enhancement was more dominant, which attracted more water molecule permeation across the membrane. The excellent dispersion of ACh nanoparticles provided more active sites for solute removal, resulting in higher salt rejection (95–97%). The hydrophilicity enhancement and surface roughness reduction for membranes with 0.5 wt.% ACh led to antifouling property improvement, as indicated by the increase in FRR from 40.4 to 80.9% after one fouling cycle using milk solution. A similar improvement in antifouling property was also observed by Khosravi et al. [124], who used chitosan to enhance the compatibility of Mil-125(Ti) nanoparticles in the PES nanocomposite. The optimal membrane performance was observed at 1 wt.% chitosan-modified MOF loading, where the highest pure water flux, highest FRR (~98% in BSA filtration), and good separation efficiency in terms of dye, antibiotics, and heavy metals removal were achieved under that condition.

For effective heavy metal recovery, developing a positively charged NF membrane with hydrophilic properties is essential to ensure high cations rejection and flux as well as low fouling tendency. For that purpose, hyperbranched polyethyleneimine (HPEI) with a positive charge and hydrophilic properties is a promising polymer that can modify the negatively charged PES NF membrane. Thus, Peydayesh et al. [125] grafted the MWCNT with HPEI polymer before fabricating the asymmetric PES-based hybrid NF membrane, aiming to induce the positive charge and enhance the polymer/particle compatibility. The hybrid NF membrane successfully demonstrated good antifouling properties and attained heavy metals rejection between 93.39% and 99.06%. Furthermore, the flux remains stable during the three-day filtration test, suggesting no MWCNT was leached out due to good particles and polymer interaction. Meanwhile, Rahimi et al. [123] used a facile method to functionalize the MWCNT nanoparticle with polysaccharide (β-cyclodextrin) before incorporating them into PES nanocomposite. In their work, the β-cyclodextrin was attached to the surface of MWCNT nanoparticles via a soft cutting method which involves the particles grinding with an antifouling agent and ethanol. The enhancement in membrane hydrophilicity has suppressed the foulant firm deposition on the membrane surface, reducing the irreversible fouling ratio.

Apart from asymmetric NF membrane fabrication, the polymer-functionalized nanoparticles have also been incorporated into polyamide TFC NF. Hydrophilic polymers such as polypyrrole, PEG, and zwitterions have been used to improve the nanoparticle’s dispersion and interaction in the polyamide layer and improve the antifouling properties of TFC NF [62,126,128]. Most researchers embedded the polymer-functionalized nanomaterials into the polyamide layer during the interfacial polymerization by dispersing the modified particles into PIP solution. Wang et al. [126] tried fabricating a highly efficient TCF NF with antifouling properties by blending the zwitterionic-functionalized porous organic framework (POF). The synergistic effects of POF and zwitterions improved the fouling resistance and enhanced the water flux from 24 LMH to 42.6 LMH while maintaining the Na_2_SO_4_ rejection (90.6%). The addition of POF into the TFC increased the water flux compared to the pristine membrane due to the formation of more and shorter channels for water transport. Meanwhile, the zwitterionic functionality improved the antifouling features by increasing the membrane hydrophilicity and providing the hydration layer on the membrane surface, preventing the foulants (BSA and HA) accumulation. The modification using zwitterionic-functionalized POF also resulted in a more electronegative membrane surface which can reduce the fouling via electrostatic repulsion with negatively charged foulants. Even though the membrane surface roughness increased after modification, this negative effect was not dominant in reducing the antifouling property.

**Figure 8:**
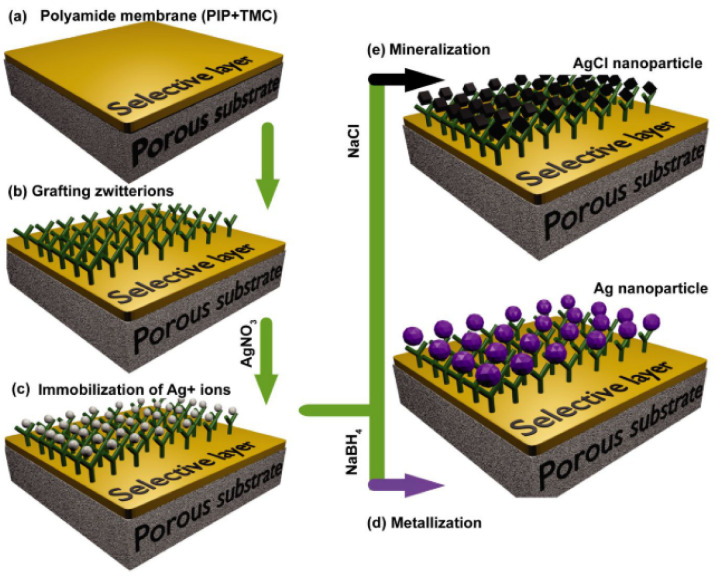
(**a**) TFC membranes comprising polyamide selective layers (yellow) and porous support (grey), (**b**) membrane functionalized with zwitterions (green), followed by (**c**) Ag ions (silver) deposition via immersion in AgNO3 solution. These membranes were immersed in different solutions to obtain (**d**) metallized membranes that contain Ag nanoparticles (purple) and (**e**) mineralized membranes that contain AgCl nanoparticles (black) [9] (Copyright 2019. Reproduced with permission from the American Chemical Society.)

Yi et al. [9] proposed another strategy to modify the TFC with zwitterionic-functionalized nanoparticles. In their work, the zwitterion was firstly grafted on the polyamide layer of TFC NF, followed by Ag nanoparticles formed in situ on the pre-grafted surface (Figure 8). The long-term anti-biofouling test which was conducted using synthetic effluent containing *S. aureus* for 12 h revealed that the TFC with zwitterionic-Ag showed a minimal decline in normalized water flux (10.5–21.6%) compared to the pristine TFC and Ag-incorporated TFC membrane (72.9% and 47.8%, respectively), suggesting the stability of the zwitterion-modified Ag complexes. Also, the regeneration of marginally clogged zwitterionic-Ag TFC membranes has recovered as much as 98% of the water flux after a long-term dynamic fouling test. Thus, it was concluded that combining Ag and zwitterions could impart both anti-microbial and anti-fouling properties without sacrificing the NF performance.

In another work, Wang et al. [127] synthesized a composite NF membrane comprised of PDA-crosslinked GO and zwitterionic polymer. Unlike the previous technique [9], the PDA-crosslinked GO nanosheets were first deposited on the PES support, followed by zwitterionic polymer grafting on the surface of the GO-based membrane via a Michael addition reaction. The role of PDA was to improve the stability between two GO nanosheets, while zwitterionic polymer acted as an antifouling layer. Besides antifouling property improvement, this modified composite membrane (Z-PEI-GO@PDA/PES) also demonstrated good structural stability even after exposure to organic solvent (ethanol) at different periods (2–8 days). Compared to the GO-PES membrane, the flux and dye rejection of the Z-PEI-GO@PDA/PES membrane remained stable after organic solvent treatment, suggesting that the PDA adhesion improved the GO immobilization on the substrate and enhanced GO sheets’ interfacial compatibility.

## 4. Challenges and Perspectives

Most of the NF membranes’ modification using various antifouling modifiers have successfully improved the fouling resistance and separation performance by tuning membrane properties such as hydrophilicity, surface roughness, surface functionality, and charge. In spite of the improved antifouling capability, several challenges need to be addressed in future work.

Table 4 summarizes the highlights and challenges of different classes of antifouling agents. Among the materials, modification using inorganic nanoparticles and polymers has been commonly applied in developing antifouling NF membranes. In terms of nanoparticle embedment into the selective layer of membrane, particle agglomeration and poor compatibility with polymer remain major concerns. These issues commonly cause defect formation, leading to reduction of the membrane selectivity. Thus, exploring nanoparticles with great hydrophilicity, low agglomeration tendency, low cost, small particle size, good dispersibility, and compatibility with the polymer can be one of the focuses of future work. For example, CQD is a promising nanomaterial that can be further explored in developing antifouling NF membranes due to its good hydrophilicity, ultra-small size, non-toxicity, favorable polymer affinity, and good dispersibility without agglomeration [68,69,129]. Moreover, pre-modification of nanoparticles with polymer (composite polymer/nanoparticle) before incorporating them into membranes has been introduced as a strategy to improve the particle dispersion and compatibility with the membrane matrix. Even though such a method could impart the synergistic effects of both polymers and nanoparticles, extra steps and additional costs are required for preparing the composite nanomaterials, which in turn make it less practical for commercialization.

Meanwhile, membrane modification using hydrophilic polymers offers more significant commercialization potential than modification using inorganic nanoparticles. Among the polymers, zwitterionic polymer and dendrimers have recently gained considerable attention as antifouling modifiers due to their unique features and great hydrophilicity. Unfortunately, membrane surface grafting and coating using these polymers often increases the mass transfer resistance and decreases the water permeability. A recent study showed that the water permeability of NF membrane could be increased even after modification using zwitterionic polymer grafting, provided that the grafting layer thickness is within the nanometer scale [114]. The surface grafting method offers a more stable antifouling layer compared to the surface-coating technique, which possesses a disadvantage in terms of disengagement of the coating layer. Thus, more studies can be conducted in the future to develop a stable antifouling polymer layer (e.g., zwitterionic or dendrimers layer) with nanometer thickness on the NF membrane. Besides the surface grafting method, the incorporation of hydrophilic polymer into the membrane via physical blending is a promising approach to developing the antifouling NF membrane. This strategy has the potential for scaling up and does not require additional production lines and costs to post-modify the membrane. However, excessive loading of polymer additives could reduce water permeability. Accordingly, optimization in polymer additive loading should be a concern to attain the antifouling property without sacrificing the membrane selectivity and permeability.

Furthermore, it should be noted that biofouling, inorganic, and organic fouling can occur at the same time and interact with one another. As mentioned previously, some studies have shown that the dominant type of NF fouling shifted along different stages of the real NF process [33]. Thus, it can be suggested that the development of antifouling NF membranes must consider the interaction and intricacies of the different types of fouling. The selection of membrane modifier agent should consider the dominant foulant types in the feed water to be treated. As an example, phosphorylated-PEI (modified dendritic polymer), which is a promising antifouling and anti-scaling membrane modifier, can be explored since it can hinder the in situ formation of pre-nucleation clusters via phophonate-Ca^2+^ complexes formation [107]. In the case of biofouling, modifier materials that integrate contact-disruption and released-based biocidal mechanisms can be investigated to enhance the biocidal action. Further research is required to develop membrane modification approaches to combat different fouling tactics and mechanisms. Fouling tests should also be performed to take into account the complex nature of real water and wastewater applications that lead to complex fouling behavior.

Another aspect that needs to be considered is the stability and long-term performance of modified NF in actual field applications. Many studies were conducted in lab-scale or controlled environments using synthetic pollutant solutions. For instance, BSA and HA solutions have been commonly used to test the antifouling behaviors of the modified membranes. However, the complex constituents in real wastewater might discount the membrane performance. A long-term feasibility study should be conducted to offer insight into how the membrane would perform when dealing with real wastewater. Moreover, it should be noted that in the real process, the application of cleaning agents such as acid and alkaline might degrade the antifouling agents. Thus, it is essential to investigate the stability of the modified membranes upon being subjected to cleaning procedures.

Besides excellent separation performance with good antifouling property, a modification strategy should be cost-effective to promote its implementation in real applications. Some promising antifouling modifiers such as zwitterionic polymers, dendrimers, and graphene-based nanoparticles possess high production costs, restricting their large-scale application. For instance, a market survey shows that the price of PAMAM dendrimer (Merck) is almost eight times higher than that of a common hydrophilic polymer such as PVA (Merck). Hence, effective modification strategies are essential to minimize the membrane fabrication cost, especially when involving an expensive modifying agent. Surface-grafting and surface-coating techniques may have a tendency for additives wastage due to some ineffective attachment of antifouling agents on the membrane surface during membrane modification steps. Compared to these methods, entrapping the antifouling modifiers into the membrane via the physical blending technique seems more promising, especially if the membrane requires a low additive loading to boost its performance. Therefore, more studies can be conducted to explore effective modification strategies that utilize a low quantity of antifouling modifiers but can improve the membrane antifouling property and separation performance.

## 5. Conclusions

There has been a great deal of interest in developing antifouling NF membranes for water and wastewater treatment applications. This review highlights the application of various antifouling modifiers (nanoparticle, polymer, and composite polymer/nanoparticle) in enhancing the fouling resistance property of NF membranes via different modification approaches. Among the antifouling modifiers, NF membrane modification using nanoparticles and polymer additives has been commonly applied. Membrane modification using composite polymer/nanoparticle can be considered a recent strategy in developing antifouling NF membranes and requires further exploration. New advanced materials such as graphene-based nanoparticles, CQD, zwitterionic polymers, and dendrimers are promising modifying agents that have attracted attention in recent years. Most of the studies found that the application of these materials has successfully improved the fouling resistance of NF membranes by tuning the membrane properties such as membrane hydrophilicity, surface roughness, charge, and functionality. However, improvement is required to explore effective modification strategies to incorporate these materials into the membrane. The effective modification strategies that utilize a small quantity of antifouling modifiers (especially for the expensive additives) to improve the membrane antifouling property and separation performance and remain stable under long-term operation can be further investigated in the future to develop advanced NF technology.

## Figures and Tables

**Table 1:** NF modification using various types of nanoparticles.

Nanoparticle	Modification Technique	Performance	Reference
TiO_2_: ZnO	Two step co-deposition (binding) on membrane	Water permeability decreased by increasing ZnO ratio. Membrane with TiO_2_: ZnO at ratio 1:2 and 2:1 showed antibacterial activity against Bacillus subtilis.	[45]
Ag@UiO-66-NH_2_	Embedded during interfacial polymerization	At 0.03 g Ag@UiO-66-NH_2_ loading, water flux was 2-fold higher than that of polyamide membrane. FRR was 95.6% after BSA filtration and antibacterial rate beyond 95%	[46]
Goe and Mf	Blending with casting solution	At 0.5 wt.% Mf, maximum water flux was achieved. FRR of 83.4–97% after four cycles of power milk fouling experiments.	[49]
CoFe_2_O4/CuO	Blending with casting solution	Water flux increased from 12 LMH * to 34.5 LMH. FRR ~90% at 0.5 wt.% particle loading	[50]
cMWNT	Embedded during interfacial polymerization	Pure water flux (>80%) >20% improvement in antifouling properties after three cycles.	[59]
PPy-r and PPy-ox MWCNTs	Embedded during interfacial polymerization	NF with PPy-ox MWCNTs and PPY-r showed 110% and 94% higher pure water flux, respectively. PPy-r MWCNTs have 90% more fouling resistance than the raw and ox MWCNTs-modified membrane	[62]
EDA-functionalized GO	Deposition on membrane surface	50% increased salt rejection Water flux recovery of the BPPO/EDA/GO-39 membrane is 51%.	[66]
CQDs	Blending with casting solution	Membrane’s stability and water permeability enhanced by more than 85%. 40% less flux drops and 85% higher flux recovery after fouling cycles	[71]
UiO-66 and UiO-66-NH_2_	Embedded during interfacial polymerization	Flux recovery rate reached 96.82% even after three fouling cycles with the BSA solution.	[79]
ZIF	Embedded during interfacial polymerization	PA/ZIF mixed matrix TFN with nanoscale Turing-type network structures showed 3–4-fold higher water flux and high salt rejection and antifouling resistance capability.	[80]
MOF@Fe_3_O_4_	Blending with casting solution	Flux enhanced from 8.1 to 28.5 LMH. 30% improvement in relative recovery rate (increased from 47 to 77%) when filtering BSA.	[84]
Cu-BTC	Coating on membrane	Flux recovery increased from 55 to 71% (alginate) and from 61 to 70% (HA).	[87]
2D MOF (BUT-203)	Coating on membrane	Water permeance increased from 148 to 870 LMH/bar. Membranes regained 89% and 93% of fluxes when filtering BSA and HA solution.	[88]
COF	Embedded during interfacial polymerization	The PES/SNW-1 membrane attained a water flux of 117 LMH, BSA rejection of 98%, and FRR of 88.9%	[89]
Octylamino (OA)- POSS	Embedded during interfacial polymerization	Enhanced water flux from 58 LMH to 90 LMH. FRR increased to 85.0%, higher than that of the polyamide membrane (79.2%)	[92]
Surfactant-modified MMT	Embedded during interfacial polymerization	Water flux (66.02 LMH) higher than that of TFC (40.93 LMH). Flux recovery of the TFN_DDAB-MMT_ reached ~100%.	[93]
Nickel-bentonite (NBNPs)	Blending with casting solution	At 0.5 wt.% NBNPs, water flux increased from 34 to 59 kg/m^2^h. Attained 98.2% FRR.	[95]

* LMH unit represents L/m^2^⋅h.

**Table 2:** NF modification using various types of polymers.

Polymer	Modification Technique	Performance	Reference
PVA	Gutter- and coating layer-assisted strategy	Water flux slightly decreased after coating More than 80% FRR during three cycles filtration of HA	[99]
4-arm PEG methoxy	Immobilized on membrane surface	FRR of 85% during the long-term filtration test which was conducted using real textile effluent.	[102]
PEG	Immobilized on membrane surface	Higher recovery ratio (99.09%) compared to that of the unmodified TFC NF membrane (89.34%).	[103]
PEI	Green rapid coating	FRR of SA, HA and BSA aqueous solutions were increased from 81.2%, 94.8% and 89.5% (in first cycle) to 98.0%, 97.7% and 98.1%, respectively (in second cycle).	[106]
Phosphorylated PEI	Immobilized on membrane surface	Water permeability of 24.2 LMH/bar Improved the antiscaling performance	[107]
Perfluorobutylsulfonyl-functionalized PEI	Incorporated through interfacial polymerization	Permeation flux of 6.5 LMH FRR up to 99.1% and 98.0% (after BSA and HA filtration, respectively).	[5]
PAMAM	Incorporated through interfacial polymerization	CB6/PAMAM-modified membrane showed anti-fouling property in both inorganic salt and organic pollutant system.	[109]
Zwitterionic	SI-RAFT grafting	Water permeability was slightly decreased after zwitterionic grafting. FRR of 92% and irreversible fouling ratio was decreased.	[110]
PDA and zwitterionic (PSBMA)	SI-ATRP grafting	Permeance decreased by 10%. Flux recovery of 92.1% during HA filtration and exhibited superior antibiofouling with reducing 86.1% of *E. coli* colonies adhered.	[111]
P(DA-SBMA)	Incorporated through interfacial polymerization	Water permeability of 73.11 LMH. Flux recovery of 99.53% was attained during BSA filtration.	[117]
PDA/PVA	Electrospraying on membrane	High FRR (98.9%) with a 6.1% of total flux decline ratio when tested with HA. Excellent chlorine resistance property	[118]

**Table 3:** NF modification using various composite polymer/nanoparticles.

Composite Polymer/Nanoparticles	Modification Technique	Performance	Reference
Chitosan-modified AC	Blending with casting solution	Particle loading up to 0.5 wt.% has increased the water flux from 21 to 30 LMH. FRR increased from 40.4 to 80.9% after one fouling cycle using milk solution.	[122]
Chitosan-modified MOF	Blending with casting solution	1 wt.% chitosan-modified MOF loading showed the highest pure water flux and highest FRR (~98% in BSA filtration).	[124]
HPEI-modified MWCNT	Blending with casting solution	Pure water flux was enhanced by 109%. Flux recovery enhances from 72.9% to 97.2% in first cycle	[125]
Zwitterionic-functionalized POF	Incorporated through interfacial polymerization	Water flux increased from 24 LMH o 42.6 LMH. Antifouling properties was enhanced after adding zwitterionic-POF.	[126]
Zwitterionic-Ag	Zwitterionic grafting on membrane, followed by Ag nanoparticles in-situ formation.	TFC with zwitterionic-Ag showed a minimal decline in normalized water flux (10.5–21.6%) compared to the pristine TFC.	[9]
PDA-crosslinked GO and zwitterionic	PDA-GO deposition on membrane support, followed by zwitterionic grafting on GO.	Flux and dye rejection remained stable after organic solvent treatment. Flux reduction was minimized when in contact with foulants.	[127]

**Table 4:** Summary of highlights/advantages and challenges of different classes of antifouling modifiers.

Class of Antifouling Modifiers	Highlights/Advantages	Challenges
Nanoparticle	Excellent hydrophilicity and antifouling feature Excellent antibacterial properties (e.g., metal, metal oxide, and graphene-based nanomaterials). Well-known synthesis techniques Porous particles (e.g., MOF) provide additional channels for water permeation. Large surface area with tunable structure (e.g., graphene, MOF)	Agglomeration issue at high filler loading. Poor compatibility between inorganic nanoparticles with polymer matrix Nanoparticles leaching under long-term operation
Polymer	A good compatibility between the polymeric chains Excellent hydrophilicity and antifouling feature. Good resistance towards biofouling (e.g., zwitterionic polymer) Modification using dendrimers can improve membrane charge density, porosity, and stability. Biodegradable and biocompatible (e.g., PDA and chitosan).	High production cost (e.g., zwitterionic polymer and dendrimers). Complicated synthesis procedure (e.g., zwitterionic). Thick polymer coating or grafting often reduces water permeability.
Composite polymer/nanoparticle	Improve nanoparticle dispersibility Enhance compatibility between nanoparticle and polymer matrix. Impart synergistic effects of both polymer and nanoparticle, improving separation performance and antifouling property.	Additional step is required to prepare the composite polymer/nanoparticle Synthesis reproducibility
